# Protective effect of curcumin on fertility of rats after exposure to compact fluorescent lamps: An experimental study

**Published:** 2018-07

**Authors:** Naser Khalaji, Mahshid Namyari, Yousef Rasmi, Masoumeh Pourjabali, Leila Chodari

**Affiliations:** 1 *Department of Physiology, Urmia University of Medical Sciences, Urmia, Iran. *; 2 *Urmia University of Medical Sciences, Urmia, Iran.*; 3 *Department of Clinical Biochemistry, Urmia University Medical Sciences, Urmia, Iran. *; 4 *Department of Pathology, Urmia University of Medical Sciences, Urmia, Iran.*; 5 *Neurophysiology Research Center, Urmia University of Medical Sciences, Urmia, Iran.*

**Keywords:** Infertility, Compact fluorescent lamps, Curcumin, Rat

## Abstract

**Background:**

Testicular function is modified by maturational gonadostatic control highly susceptible to negative physiologic niche-altering factors like UV-rays.

**Objective:**

This study was performed in order to uncover new aspects of Compact Florescent Lamps (CFLs) induced damages on the testicular tissue of rats and evaluating the effect of curcumin on testis of rats after exposure to compact florescent Lamps.

**Materials and Methods:**

Twenty-four adult male Albino rats were randomly divided into three groups: control group (ethyl oleate 0.2 ml, IP, for 45 days, without CFLs exposure), fluorescent group (ethyl oleate 0.2 ml, IP, daily and treated with 12 hr CFLs exposure for 45 days) and curcumin group (curcumin 20 µ M, IP along with 12 hr CFLs exposure for 45 days). The rats were anesthetized at the end of the experiment. Gonadotropin hormones and prolactin levels were measured; Histopathological and histomorphometrical analysis of the testis was carried out.

**Results:**

Results of this study showed that CFLs significantly decreased serum levels of follicle stimulating hormone, prolactin, testicular weight, sperm motility, TDI, and SPI. Furthermore, CFLs had no effect on serum levels of luteinizing hormone and sperm count and also, increased abnormal sperm shapes. Our results also showed that curcumin supplementation following CFLs reversed these alterations.

**Conclusion:**

These results strongly suggest that CFLs severely impairs testis while curcumin as an antioxidant had protective effects on undesirable effects in testis induced by CFLs.

## Introduction

Infertility is regarded as a social problem amongst all cultures and societies. It affects about 10-15% couples in reproductive age  (1). Around 60% of all infertile couples exhibit male infertiliry factors. The regulation of spermatogenesis includes together endocrine and paracrine mechanisms (2). follicle stimulating hormone (FSH) and luteinizing hormone (LH) are both the endocrine stimulation of normal spermatogenesis (2). It has been previously reported that infertile and azoospermic males have a higher or lower the serum levels of both LH and FSH  (3).

Another important factor in the male fertility is sperm morphology. It is reported that abnormalities in sperm morphology reduce male reproductive potential (4). In the most recent report, there are environmental factors along with lifestyle practices that contribute to the deterioration of semen quality (5). Exposure to high levels of ultraviolet radiation (UVR) is one such type of environmental stressor that can have adverse effects on the genital system and reproductive performance (6). One of the most significant sources of UV radiation is compact fluorescent lamps (CFLs) (7). However, a few studies have focused on the effect of UV radiation on male infertility include hormonal changes, histology of testes and quality and quantity of spermatogenesis. 

In recent years, much attention has been focused on the use of active dietary ingredients, such as phytochemicals in remedial different diseases related to oxidative stress (8). Curcumin is a yellow powder that is derived from the rhizome of turmeric. Curcumin has a broad spectrum of biological and pharmacological activities (9). In addition, the antioxidant and anti-inflammatory properties of curcumin, is well known (10). It is shown that curcumin has stimulatory effects on the reproductive system and could protect the testes from the toxic effects of gallic acid (11). Similarly, it is reported that curcumin improves sperm parameters (percentages of motility, sperm motion characteristics, acrosome and total abnormalities) in frozen-thawed bovine semen  (12). 

The aim of this study was to investigate the effect of curcumin on FSH and LH hormones, quality, and quantity of sperm and testicular tissue.

## Materials and methods

**Animals**

Twenty-four adult male Albino rats (230±30 gr body weight) were randomly divided into three groups:

- The control group (ethyl oleate 0.2 ml, IP, for 45 days, without CFLs exposure).
- Fluorescent group (ethyl oleate 0.2 ml, IP, daily and treated with 12 hr CFLs exposure for 45 days).
- Curcumin group (Curcumin 20 µmol 0.2 ml, IP along with 12 hr CFLs exposure for 45 days).

**UV-irradiation**

The animals were placed in boxes covered with aluminum sheets and expose to one CFLs (40 w) for 8 hr per day. The size of each box was one square meter. The CFLs were put at a distance of 10cm from rats emitting essentially at 280-400 nm, divided into two distinct spectral areas including ultraviolet A and ultraviolet B rays (13). The intensity of each lamp was UVA (1.06 W/m^2^) and UVB (0.02 W/m^2^). Twenty-four hr after end of UV exposure, the animals were anesthetized with pentobarbital (40 mg/kg, IP). 

Then, testes and epididymis were removed and immersed into 10% formalin after excision. Blood samples were collected for biochemical analysis. 

**Chemicals**

Curcumin (Sigma Company for Chemicals, Iran) dissolved in ethyl oleate and administered subcutaneously to rats in the curcumin group at 20 µmol dose once daily for 45 days (14). 

**Measurement of hormones**

Serum FSH, LH, and prolactin levels were estimated by immuno enzymatic assay by Elisa Reader. The kits were obtained from DRG Company, Germany.

**Sample collection**

Orchidectomy was performed by open castration method. A horizontal incision was performed in the scrotum, the testes were tied off and removed and in tunica vaginalis. Semen samples were collected from the cauda epididymis. The methods of collection were similar to that described by Akusu and co-worker (15). The samples were analyzed immediately after collection.

**Sperm density, Motility, and Morphology**

The mature spermatozoa were collected from the cauda of the right epididymis by mincing it finely in Tyrode's buffer solution to a final volume of 3.0 mL at 37^o^C. A sample of this sperm suspension was immediately placed on a pre-warmed hemocytometer to determine motility by counting all sperm in 20 fields (×40). A 1.0 µL portion of sperm suspension was incubated with eosin-nigrosin, and a fine smear was examined under light microscope (×1000) to evaluate sperm morphology, with classification of sperm as normal or abnormal (no hook, excessive hook, amorphous, pin-head, two heads or two tails, and short head) as described by Wyrobek and Bruce (16). To determine sperm density, a sample of sperm suspension was heated in boiling water bath for 30 sec (killing all sperm) and counted with a hemocytometer.

**Histological analysis**

After 45 days, the testicles were dissected out and fixed in Bouin's solution for 1 wk. Samples were processed through paraffin embedding and the blocks were cut by a rotary microtome (MICROM GmbH, Germany) and stained with hematoxylin-eosin (H&E). Two hundred cross sections of seminiferous tubules were randomly analyzed (one hundred per testis) for calculation of tubular differentiation index (TDI) and spermiation index (SPI).

**TDI**

To estimate TDI, the percentage of seminiferous tubules with more than three layers of differentiated germinal cells from spermatogonia type A were counted and considered as TDI positive (17).

**SPI**

To estimate the SPI, the ratio of the number of seminiferous tubules with spermatozoids to the empty seminiferous tubules were calculated and considered as SPI positive (17). 

**Ethical consideration**

All animal experiments were performed under the guidelines on the human use and care of laboratory animals for biomedical research published by National Institutes of Health (8^th^ ed., revised 2011) and conformed to the Declaration of Helsinki. The Ethics Committee of Urmia University of medical science approved the experimental protocol (Ethics Committee code: IR.UMSU.REC.1395. 203).

**Statistical analysis**

All data are expressed as mean±SD and the difference for comparison was considered significant at p<0.05. For statistical analysis SPSS package (Statistical Package for the Social Sciences, version 18.0, SPSS Inc, Chicago, Illinois, USA) was used for one way analysis of variance (ANOVA) and Tukey post-test.

## Results

**Effect of curcumin on LH, FSH and prolactin serum levels**

**Figure 1:**
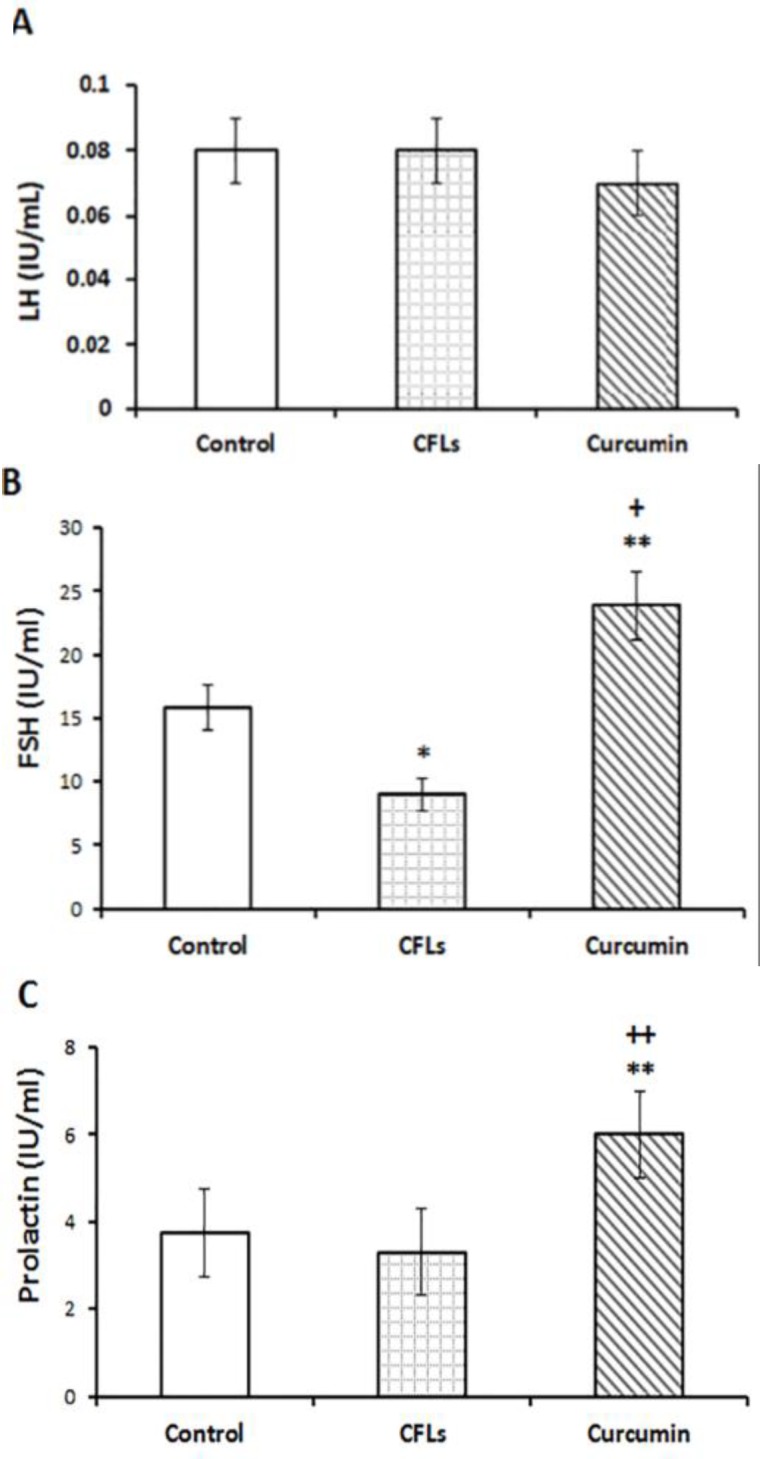
Comparison of the serum levels of LH (A), FSH (B) and Prolactin (C) in rats with and without exposure to compact fluorescent lamps or treatment with curcumin for 45 days. Data are presented as means ± SD for 7 animals. *p<0.05, **p<0.01 vs. the control group. ++p<0.01 vs. the CFLs group

Figure 1A shows that after 45 days of treatment of rats in CFLs group with curcumin had no significant change in LH serum levels compared to the CFLs group. Also, a comparison between the control groups with CFLs group exhibited no significant difference among these groups. Figure 1B illustrates that CFLs group significantly (p=0.032) had lower FSH levels in theirs serum compared to the control group. After treatment of CFLs group with curcumin, the level of FSH in serum were significantly increased in comparison with control (p=0.024) and CFLs (p=0.0053) groups. Figure 1C shows that the level of prolactin in the serum of CFL group was lower than the control group, but the difference was not significant. In the curcumin group, 45 days treatment with curcumin significantly (p=0.0062) increased prolactin levels in serum in comparison with the control and CLF groups.

**Effect of curcumin on the total sperm, sperm motility, sperm mortality and testicular weight**

**Figure 2:**
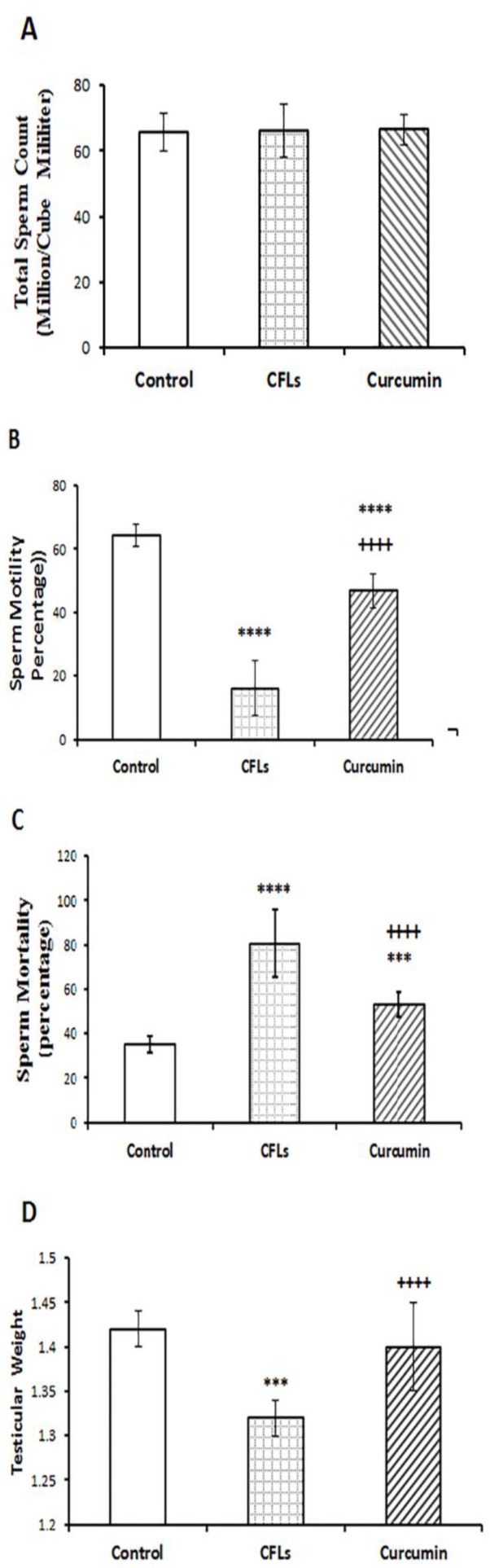
Comparison of the total sperm (A), sperm motility (B), sperm mortality (C), testicular weight (D) in rats with and without exposure to compact fluorescent lamps or treatment with curcumin for 45 days. Data are presented as means ± SD for 7 animals. ***p<0.001, ****p<0.0001 vs. the control group. ++++p<0.0001 vs. the CFLs group

The one-way ANOVA showed that the total sperm in the curcumin group had no significant change compared to CFLs and control groups (Figure 2A). As shown in figure 2A, treat rats with CFLs in the CFLs group total sperm count compared to the control group. Figure 2B shows that sperm motility significantly (p=0.043) decreased in CFLs group compared to the control group. In curcumin group, sperm motility significantly (p=0.033) increased compared to CFLs group but it was still significantly lower than that of the control group (p=0.092). Figure 2C shows that sperm mortality significantly (p<0.054) increased in CFLs group compared to control group whereas in curcumin group, sperm mortality significantly decreased compared to CELs group (p=0.087) but it was still significantly higher than that of the control group (p=0.034). Figure 2D shows that testicular weight significantly (p=0.065) decreased in CFLs group compared to the control group. In *c*urcumin group, testicular weight significantly (p=0.045) increased compared to CFLs group.

**Effect of curcumin on sperm morphology**

**Figure 3:**
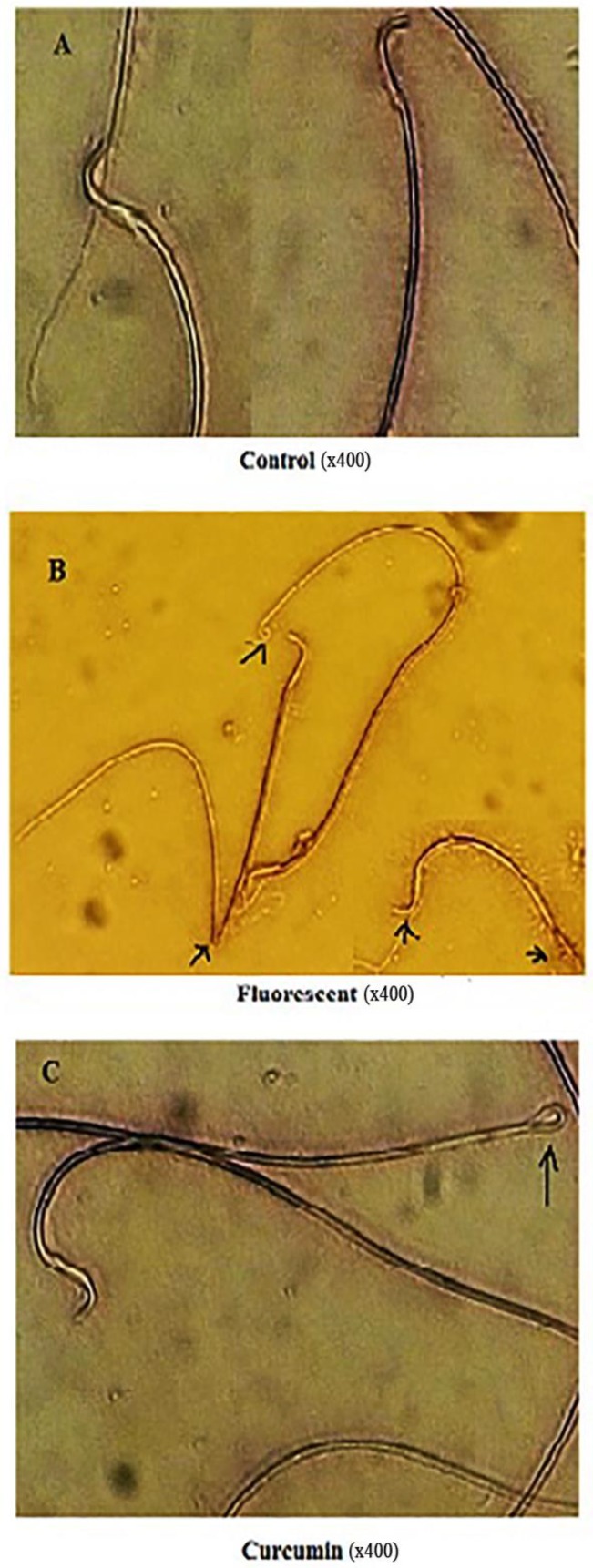
Morphology of spermatozoa in adult male rats (40×10). Arrows show abnormal sperm with head and tail defects

Study of morphology of spermatozoa of control rat showed characteristic sickle-shaped head and normal shape of trunk and tail whereas in CFLs group, 64 percent of all counted spermatozoa were abnormal and it was also statistically significant (p=0.079). As shown in Figure 3, abnormal spermatozoa are visible with double-headed, halved trunk and short and twisted tail. In curcumin group, the number of abnormal sperm was 21% of the total sperm counted and significantly lower than CFLs group and there is partly cellular integrity but immature spermatozoa are observed in the seminal tubes (Figure 3).

**Effect of curcumin on testicular tissue morphology**

**Figure 4:**
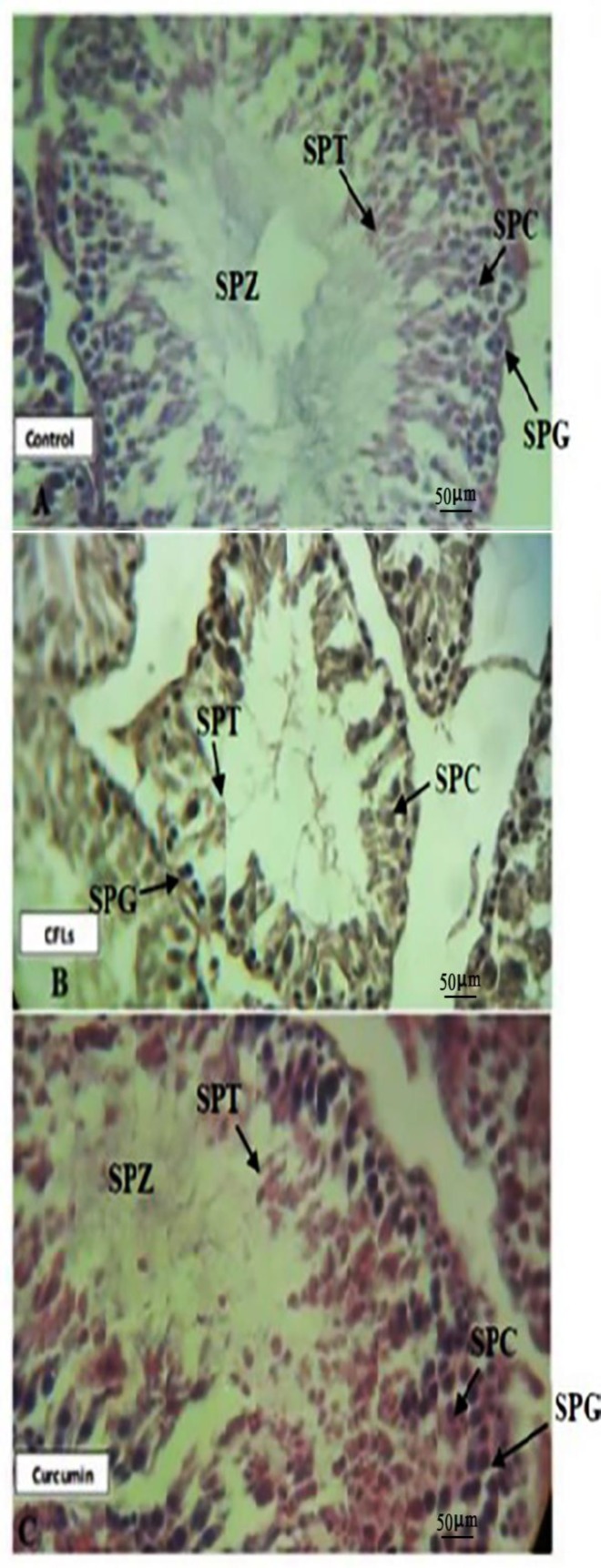
Cross section from seminiferous tubules

As shown in Figure 4A, in the control group, cellular layers of the walls of the seminiferous tubules are normal and their cells are arranged. Spermatogonia, primary spermatocytes, secondary spermatocytes, spermatids, spermatozoa, and Sertoli cells are observed clearly and naturally. Figure 5B shows that in the CFL group, the cellular consistency of the seminiferous tubules has been lost. Also, TDI and SPI significantly (P=0.065) decreased compared to the control group (Table I). According to the Figure 4C, in curcumin treated group, walls of the seminiferous tubules are fairly arranged and cellular consistency has been partly preserved. Also, the mean value of tubular differentiation (TDI) and spermiogenesis index (SPI) was significantly (p˂0.001) higher compared to the CFL group (Table I).

Comparison of the TDI and SPI in testis of rats with and without exposure to compact fluorescent lamps or treatment with curcumin for 45 days.

**Table I:** Data are presented as means±SD for 7 animals

**Groups**	**SPI (%)**	**TDI (%)**
Controls	90.3 ± 3.5	95.4 3 ± 2.5
CFLs	50.1 ± 6.0#	48.20 ± 6.4#
Cucumin	86.7 ± 4.6$	81.50 ± 7.5*$

TDI: Tubular differentiation index.

SPI: Spermatogenesis index

* p<0.05 and

# p<0.0001 vs. The control group.

$ p<0.001 vs. The CFLs group.

a) Control group showing normal morphology of head , trunk and tail of sperm

b) CFLs group showing decapitation in head portion and short and twisted tail

c) curcumin group showing partly integrity and Immature spermatozoa are observed in the seminiferous tubules. Eosin-Nigrosin staning.

A) control group showing normal seminiferous tubules with active spermatogenesis and normal Leydig cells

B) CFLs group showing shrinkage of seminiferous tubules, significant decrease in the number of spermatogonia, spermatocytes and spermatids and complete absence of spermatozoa the germinal epithelium also showed degenerative changes and

C) curcumin groups. H&E staining. (×400). SPG, spermatogonia; SPC, spermatocytes; SPT, spermatids, SPZ, spermatozoa.

## Discussion

The present study was designed to determine the protective effects of curcumin against testis damage in animals after exposure to CFLs. To accomplish this, we compared the responses in animals with CFLs exposed with those of male rats with CFLs exposed that were treated with curcumin. Our findings showed that the levels of serum FSH and prolactin were significantly decreased after exposure to CFLs. Exposure to CFLs in rats had an adverse effect on sperm mortality, sperm motility, and testicular weight. Our finding showed that treatment rats with curcumin reversed these adverse effects of CFLs. Morphological study of spermatozoa was acknowledged significant increase in the number of degenerative forms and decrease in the TDI and SPI after exposure to CFLs. After treatment with curcumin, this undesirable effects of CFLs on testicular tissue morphology and spermatozoa morphology was reversed. 

Many types of lighting sources used in our homes emit small amounts of UVR. CFLs emit slightly more UVR when compared to other light sources like halogen and incandescent light sources. The UV-irradiation from CFLs has recently become a “new vector of aberrant human physiology  (18). In this study, it was shown that exposure to CFLs significantly decreased the levels of serum FSH and prolactin and had no effect on the levels of serum LH. Falkenbach and colleagues reported that exposure to suberythematous doses of UV did not influence the regulation of pituitary hormones in these healthy individuals (19). While it is shown that acute exposure to UV-rays potently decreases the levels of serum FSH and prolactin in rabbits (20). These findings are consistent with evidence in the literature on the toxicity of UV-irradiation (21). 

As, it is shown that, UV irradiation induces the formation of ROS in organisms that results in oxidative stress (22). Therefore, it is likely that oxidative stress plays an important role in reducing the levels of serum FSH and LH in UV-expose rats. Also, the findings of this study especially demonstrated that curcumin supplementation alleviated the extent of UV-induced suppression of the level of circulating FSH and prolactin. Similar to our work, it is shown that curcumin increase FSH and LH levels in cisplatin-treated rats (23). Also, Sadoughi reported that curcumin increases FSH and LH levels in diabetic male rats (24). Furthermore, it revealed that curcumin increases prolactin levels in women with Premenstrual premenstrual Syndrome (25). 

In general, curcumin known as antioxidant and anti-inflammatory agent that exhibit various biological effects such as anti-humoral, anti-ischemic and anti-hepatotoxic activities (26). It is evident that FSH stimulates the conversion of spermatogonia to spermatocytes and also maintains the spermatogenic process. Both FSH and LH are necessary for meiosis, formation, and development of spermatid (27). It is reported that a reduction in the availability of FSH and LH reduced the number of spermatogonia, spermatocytes and spermatid in the testis (28). Also, it is shown that prolactin acts on the Leydig cells to increase their responsiveness to LH and prolactin plays a regulatory role in spermatogenesis (29). Furthermore, it is shown that androgen is produced under the influence of LH when prolactin is also present (30). In the present study, it seems that the high morbidity of the sperm with a reduction in their motility and an increase in abnormal sperm in rats with CFLs exposure, due to the reduction of FSH and prolactin serum levels in this group. In line with our work ultraviolet C irradiation decrease human sperm motility and viability (31).

Also, it is shown that X-rays cause apoptosis in the sexual cells (32). As well as, it has been shown that UV ray prevents the molecular and cellular mechanisms of sexual growth in the early stages of life (33). Researchers have shown that high levels of ultraviolet rays have a delayed effect on the retention and production of reproductive cells (34). Our study is consistent with the above-mentioned research. The human semen is believed to contain different cell types including sperm cells at various stages of maturity, epithelial cells, leukocytes and others. It is indicated that UV rays stimulate the production of reactive oxygen species (ROS) by leukocytes, especially neutrophils and macrophages (35). When produced in large amounts, ROS have a potential toxic effect on sperm quality and function. Indeed, recent reports indicate that high levels of ROS are detected in the semen samples of 25-40% of infertile men    (36). ROS increases sperm mortality by reducing the quality and quantity of semen and increasing cell permeability in sperm (37). Furthermore, ROS interrupt mitochondria functions, synthesis of DNA, RNA, and proteins, increase DNA fragmentation, adjust the cytoskeleton, and affect the axoneme, through the oxidative stress and the making of cytotoxic aldehydes (38). DNA damaged can affect sperm roles like motility by declining the ATP required for the sperm motility. Also, lipid peroxidation reaction consequences in changes in sperm membrane fluidity, loss of membrane integrity as well as irreversible loss of sperm motility (39). In this study, interestingly, exposure to CFLs in rats had no significantly effect on sperm count. It is maybe due to the longer duration of rats spermatogenesis (52 days) is than our treatment duration (45 days) (40). Also, our result showed that curcumin reduces the mortality rate of sperm in CFLs group. 

Many studies reported that curcumin decreases MDA and oxidative stress in various tissues (-). So, it can be assumed that curcumin, as an antioxidant, inhibits reactive oxygen and induces antioxidant responses in the cell and thereby reduces the adverse effects of UV-rays.

## Conclusion

In conclusion, the results of this study have explicitly demonstrated that CFLs light seriously impacts testicular function negatively. It caused tissue changes in the testicles of male rats, reduced testicular weight, increased sperm mortality, reduced sperm mobility and decreased follicle-stimulating hormone. Curcumin, as an antioxidant, potently ameliorates this CFLs effects.

## Note

***This article extracted from M.D. Thesis. (Mahshid Namyari)***
